# Don’t Know Much about Bumblebees?—A Study about Secondary School Students’ Knowledge and Attitude Shows Educational Demand

**DOI:** 10.3390/insects9020040

**Published:** 2018-04-10

**Authors:** Anne-Kathrin Sieg, Rudolf Teibtner, Daniel Dreesmann

**Affiliations:** Department of Biology Education, Institute of Organismic and Molecular Evolution, Johannes Gutenberg University, D-55099 Mainz, Germany; rteibtne@students.uni-mainz.de (R.T.); daniel.dreesmann@uni-mainz.de (D.D.)

**Keywords:** Bumblebees, *Bombus*, pollinators, insects, knowledge, attitude, biology education, biodiversity, conservation, environmental awareness

## Abstract

Many insects are threatened with extinction, which in the case of pollinating insects could lead to declining pollination services and reduced ecosystem biodiversity. This necessitates rethinking how we deal with nature in general. Schools are ideal places in which to instill a willingness to behave in an environmentally-friendly way. Whereas scientific studies and school textbooks stress the importance of honeybees as pollinators, the role of bumblebees is either underestimated or neglected. The aim of this study was to provide information concerning student knowledge and attitudes, which are important factors of an individual’s environmental awareness. A questionnaire with closed and open questions was developed, which also included drawing and species identification tasks. We surveyed 870 German secondary school students between 9 and 20 years of age. Our results indicate limited knowledge of bumblebees by students of all grades. Knowledge increased with higher grades but only with a small effect size. The attitude of students towards bumblebees was generally positive; however, this positivity declined with increasing grade of the participants. This correlation also had a small effect size. Our results are discussed, with a particular focus on future educational demand.

## 1. Introduction

### 1.1. Loss of Insect Biodiversity—A Worldwide Threat

Recently, the widespread decline of insect species has elicited considerable public attention [1,2]. In addition to the loss of biodiversity, the absolute number of insects is also radically declining if one considers their biomass per area [2]. This impacts an ecosystem in several ways. Insects transform biomass and influence the carbon and nitrogen cycling [3]. As pollinators, insects contribute not only to an ecosystem’s biodiversity, but also tremendously to agricultural productivity [1,4,5]. Overall, insects are a crucial component of the food web, which is why their availability influences other animals such as birds [6].

To the general public, the western honeybee (*Apis meliferra*) is considered to be *the* pollinator. However, managed honeybee stocks have declined and the well-documented death of thousands of honeybee colonies in e.g., Europe and North America is alarming [7]. According to recent studies, 70% of the 124 most valuable crops for human consumption rely on bees as pollinators [8]. However, the demand for crop-pollinating insects has increased. Therefore, the role of wild-pollinating insects such as solitary bees, wasps and bumblebees as alternative pollinators has recently received more attention, primarily to compensate for failing pollination services and to cope with yield decreases [9], but also through gardening tips for the general public. In fact, the species richness of wild bees and other pollinators has also decreased over the past 50 years and some species have even become extinct [10,11]. Taking this into consideration, bumblebees have become economically relevant [1,12] and are recommended for the commercial production of tomatoes, blueberries or cranberries, among other species [13].

### 1.2. Loss of Insect Biodiversity—An Educational Challenge

To tackle the problems mentioned above, it is clear that people should know more about insects, their benefits and how to protect them [14,15]. Moreover, knowledge about biological taxa or a particular species is relevant if Senegalese politician Baba Dioum’s famous quip from a 1968 speech to the General Assembly of the International Union for the Conservation of Nature and Natural Resources (IUCN) is taken seriously: “In the end we will conserve only what we love, we will love only what we understand, and we will understand only what we are taught” [16]. As schooling represents a period of life where not only biological content knowledge is acquired, but also rethinking processes, e.g., how to behave in an eco-friendly manner, are initiated, biological lessons are also an appropriate milieu for environmental education. Teaching can help to acknowledge environmental problems, in order to change the way that people think and act. Experiences of nature during childhood considerably affect people’s perception of nature for the rest of their life [17] (p. 110). In relation to the loss of insect biodiversity, students need to learn about insects in biology classes to create an awareness of them in society and to generate a willingness to act in an environmentally friendly way.

Honeybees are extensively dealt with in German teaching materials: they are used as an example of a pollinator, to illustrate insect morphology, development, and to outline their role in an ecosystem, for example as eusocial insects, to name a few examples [18,19,20,21,22,23,24,25,26]. However, because honeybees are also associated with fear [27,28], they do not receive much conservation support. In contrast, other pollinating insects such as bumblebees are highly underrepresented, although most students seem to be familiar with them. This is rather surprising, considering that many compatible topics are taught as part of German high school biology curricula, such as those used in the federal state of Rhineland-Palatinate. For example, pollination, biodiversity, insect morphology, taxonomy, ecology, environmental protection, superorganisms and social insects are listed as obligate or optional topics in various secondary school curricula ranging from grade five, which is the first year of secondary school, to grade thirteen, which is the year prior to secondary school graduation [29,30,31].

### 1.3. Student Knowledge and Attitudes

The willingness to behave in an environmentally friendly way is influenced by knowledge and attitude [14,15,32], which can also affect each other [33]. 

#### 1.3.1. Knowledge

Conservation-oriented knowledge emerges if people understand external factors, such as ecological relationships and connectivity [14]. Additionally, knowledge concerning resource limitations promotes conservation-oriented practices in everyday life [14]. Several studies have shown that little knowledge about insects exists [34,35,36]. Research on student knowledge has often focused on invertebrates or insects in general [36,37,38,39,40]. Other studies have focused on taxa such as beetles, butterflies or ants [34,41,42,43]. Focusing on public knowledge about bees, Wilson, Forister and Carril found out that people are largely uniformed [44]. However, research on student knowledge of bumblebees is lacking.

#### 1.3.2. Attitude

In addition to knowledge, attitudes towards nature in general and to particular taxa or species, is another influential factor that also affects the willingness to act in an ecofriendly way [15,32]. Attitude is defined ‘as an overall evaluation of an object that is based on cognitive, affective, and behavioral information’ [45] (p. 4). Attitude is an hypothetical construct that cannot be directly observed, but has to be inferred from measurable responses [46]. Ajzen [46] (p. 4) discusses three categories from which attitudes can be inferred. The first is termed cognition, which is verbalized through expressions of beliefs and thoughts about the attitude object. The second category of attitude is called affect, which is verbalized through expressions of feelings towards the attitude object. The final category of attitude is conation, which is verbalized through expressions of behavioral inclinations and intentions [46] (p. 5). Insects are often associated with fear and disgust [33,37,47,48], which can impede the success of environmental education [49]. Especially honeybees are associated with danger by students. However, students’ attitude towards honeybees is positive in general [48]. Animals that are perceived as to be feared do not receive much conservation support [50]. Therefore, the attitude towards an animal influences the willingness to support it [32]. 

In conclusion, we recognize that it is important for students to develop a positive attitude towards bumblebees if we want students to protect them. Therefore, an awareness of attitude and knowledge towards bumblebees is imperative, to be able to appropriately design courses. To date, such research on student attitude towards bumblebees is lacking.

### 1.4. Learning by Accumulating Experiences of Nature

How can students’ knowledge and attitude be affected? Kellert [51] investigated learning during childhood in relation to nature experiences. He classified potential experiences of nature into three modes: direct, indirect and symbolic experiences. Direct experiences involve physical contact with plants or animals in their natural environment [51] (p. 118), whereas indirect experiences involve physical contact in a man-made environment such as an aquarium or zoo. Symbolic experiences are not gained by physical contact, but via media, e.g., books or films [51] (p. 119). Kellert concludes that direct, indirect, and symbolic experiences all affect the development of children’s learning [51] (p. 120) and he particularly emphasizes the importance of direct experiences [51] (p. 138). The dominance of electronic media, urban sprawl, and biodiversity loss, means that direct experiences of nature by children are decreasing [51]. However, the ‘direct experience of nature plays a significant, vital, and perhaps irreplaceable role in affective, cognitive, and evaluative development’ [51] (p. 139). Because the modern world has become appreciably less connected with nature, it is important to teach nature in an outdoor context [51,52]. Other studies support this assumption [53]. Their results suggest that outdoor experiences influence environmental attitude and knowledge of conservation. In addition, long-lasting effects of knowledge and attitude could be illustrated. Wüst-Ackermann et al. [38] found that an out-of-school intervention is more effective than an equivalent lesson in school.

### 1.5. The “Hallo Hummel” Project

The work presented here is within the scope of a newly established educational research project, which will integrate buff-tailed bumblebee (*Bombus terrestris*) colonies into the biology lessons of German high schools. Although honeybees are frequently kept on school grounds with or without the help of local beekeepers, teachers are rather unfamiliar with establishing bumblebee colonies. However, raising bumblebees in artificial nest boxes is fairly easy and queens can be caught and transferred to them in early spring once they have completed hibernation [54]. Moreover, there are a lot of compatible topics in the curriculum of Rhineland-Palatine, for example pollination, biodiversity, morphology of insects, taxonomy, ecology, environment protection, superorganism, or social insects [29,30,31]. By working with live colonies, the aim of the so-called ‘Hallo Hummel’ (engl. ‘hello bumblebee’) project is to develop innovative teaching materials on biological issues with the help of bumblebees as an attractive flagship species for secondary school biology classes. Besides gaining knowledge through hands-on activities, a focus will be the demonstration of protective measures that each student might potentially implement in everyday life, to counteract bee and insect extinction and to protect biodiversity.

Our study design follows that of a previously conducted research project, which implemented ant research as a new topic for schools by bringing live ant colonies into the classroom. In the so-called A.N.T.S. project, we demonstrated that hands-on investigations with living ants are more motivating for students than alternative approaches such as educational videos [55]. Moreover, we showed that direct observations in the field as well as the classroom significantly improved student knowledge and attitudes [56]. For the A.N.T.S. project, we chose a quantitative approach to collect sufficient data [34] as a basis for the development of teaching units [57].

### 1.6. The Present Study 

As to ants, research on student knowledge and attitudes concerning bumblebees is lacking. Therefore, the present study investigated student knowledge of bumblebees and attitudes towards them. 

The specific research questions of this study were:

(1) Are students familiar with the biology of bumblebees (morphology, ecology and agricultural importance, colony life)?
(2) Are students capable of differentiating bumblebees from other insects depicted in photographs?
(3) What positive or negative attitudes towards bumblebees do students have?
(4) Do differences exist between different grades with regard to student knowledge and attitudes?

## 2. Materials and Methods

### 2.1. Participants

In our survey from the end of August to November 2017, the questionnaire was completed by *N* = 870 students from Germany. The approval of the state school authority to conduct the survey was granted on 28 August 2017. The participants were students from three different schools and six different class levels in the metropolitan area of the Rhine-Main region (Table 1). We addressed several high schools to generate our sample. The biology teachers of several classes agreed in being part of our study. As a result, we have different numbers of students of each grade. We excluded data of grades ten and 13 from all grade-dependent analyses because sample sizes were small (see Table 1). However, we kept these students included in the sample for the verification of the questionnaire and for average results. Using a closed question format, we asked personal information about the participants. In total, 422 male and 404 female students formed part of the assessment. All participants had regular biology lessons and no special teaching units as preparation for the survey. Students completed the survey voluntarily and independently during class. They were asked not to cheat and to fill out the questionnaire carefully. The answers did not influence the students’ grades and questionnaire completion took a maximum of 20 min.

### 2.2. Questionnaire

We used a paper-pencil-test to collect data from the participants about their attitude and knowledge about bumblebees. We focused on aspects that we considered relevant for teaching modules. Knowledge questions were oriented towards knowledge books for young people and popular scientific literature [58,59,60]. The knowledge questions were tested in a pilot study prior to the start of the study. We applied the construct of face validity. We structured 35 questions concerning knowledge about bumblebees in four categories:

1. Morphology (six questions and one task where students were asked to draw the legs of a bumblebee at the correct positions)
2. Ecology and agricultural importance (seven questions)
3. Colony life (nine questions)
4. Species identification (twelve tasks)

In the category ‘species identification’, we presented color pictures of wasps, flies and bees and asked the participants to decide whether they represented a bumblebee or not. In the presented pictures, the number or point of origin of the legs could not clearly be seen; therefore, students could not subsequently sketch the legs correctly by looking at the pictures.

We also measured attitude by a five-tier Likert-scale (‘agree’, ‘rather agree’, ‘am undecided’, ‘rather do not agree’, ‘do not agree’). According to the components of attitude of Ajzen [46], we provided statements about bumblebees, which we classified into three factors:

1. Cognition
2. Affect
3. Conation

The chosen items are listed in Table 2.

### 2.3. Data Analyses

We evaluated the data with SPSS (Version 23.0) following Field [61]. Descriptive statistics were used to analyze knowledge and attitude with respect to grade level. 

To evaluate knowledge, we counted the correctly answered items; items that were answered with ‘do not know’ and wrong answers, were combined. We evaluated the mean percentage of correct answers for each category (‘morphology’, ‘ecology and agricultural importance’, ‘colony life’, ‘species identification’) and for the total knowledge score.

We used a principal axis factor analysis to evaluate the validity of the attitude scale and to generate categories. Furthermore, we analyzed Cronbach’s α for the three components and the complete attitude. We assigned 1 to ‘do not agree’, 2 to ‘rather do not agree’, 3 to ‘am undecided’, 4 to ‘rather agree’, and 5 to ‘agree’. An increase in the numeric value represents a stronger approval of the statement and a more positive attitude towards bumblebees. Items that express a negative attitude (see (−), Table 2) have been inverted for analysis.

We used non-parametric tests because the collected data did not follow a normal distribution. The Mann–Whitney test was used to compare knowledge and attitude between two grade levels. To examine relationships between grade level and attitude or knowledge, we used the Spearman’s correlation. Furthermore, we calculated an effect size for the significant results. The results are reported based on the recommendations of Field [61].

### 2.4. Verification of Reliability and Validity of the Attitude Questionnaire

Since to our knowledge, no questionnaire exists to systematically survey attitude towards bumblebees, we had to develop the questionnaire ourselves. We decided to measure attitude by a five-tier Likert-scale. To check the validity, we performed a principal component analysis.

The principal axis factor analysis was conducted on 21 items with Varimax rotation [61]. Four items were removed because they loaded highly by two factors without a logical explanation being possible. For example, we left out the item ‘If I spot a bumblebee in my room I would watch it carefully’, because we obtained a high loading on the category affect. In the situation described, the students’ compassion for the bumblebee might have been greater and more logical in conjunction with a positive attitude, rather than the desire to observe the animal closely.

Therefore, the PCA (principal axis factor analysis) with Varimax rotation was repeated with 17 items. The Kaiser–Meyer–Olkin measure verified the sampling adequacy for the analysis, KMO = 0.901 (‘marvellous’ according to [62]). Although PCA indicates the presence of four factors with eigenvalues greater than 1.0, a three-factor solution was chosen based on the screen plot and theoretical considerations. The principal axis factor analysis with Varimax rotation and three preset factors was conducted again. Three preset factors explained 55.83% of the variance. Table 2 shows the factor loadings after rotation. The items that clustered on the three factors can be interpreted as the cognitive, affective, and behavioral components of attitude [45,46]. Item V10, V15 and V17 loaded highly by two factors and therefore, the classification of these items into the subscale was implemented logically.

All subscales of attitude, cognition, affect, and conation had high reliability scores (Table 2). The complete attitude towards bumblebees also had a high reliability with Cronbach’s α = 0.90.

## 3. Results

To the best of our knowledge, this is the first systematic investigation of student knowledge and attitude towards bumblebees.

### 3.1. Knowledge

#### 3.1.1. Knowledge in Total

Student knowledge about bumblebees was measured by 35 questions in four categories. The knowledge scores were low for all grades. The means for different grades differed only slightly (see Table 3).

On average, the students answered fewer than half of all questions correctly *(M* = 48.01%; *SD* = 13.5%). The grade of the students was significantly correlated with the complete knowledge about bumblebees (*r* = 0.17, *p* < 0.001).

Student knowledge was ascertained by several questions. Examples are shown in Table 4.

#### 3.1.2. Morphology

In the category ‘morphology’, students (*N* = 867) answered 28.74% (*SD* = 17.72%) of the questions correctly. A large proportion of students (78.2%; *N* = 852) did not know that a bumblebee can sting to fend off an enemy (M1, Table 4); 41.7% of students thought that bumblebees can only bite; 21.8% of participants gave the correct answer; 12.1% of participants stated that they did not know what is true and 3.3% of students thought that bumblebees could neither bite nor sting.

**Figure 1:**
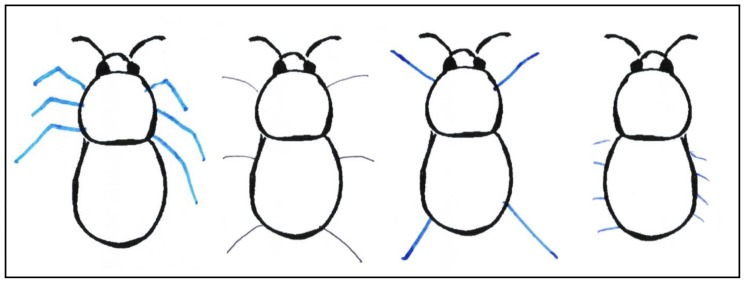
Selected examples of the legs drawn on the bumblebee body by students. The illustration on the far left shows an example that was rated as correct. The second illustration from the left shows the most common task solution of students. The two illustrations on the right represent alternative common sketches of bumblebee legs.

Furthermore, an extreme lack of morphological knowledge is illustrated by task M2 (Table 4) (Figure 1). Participants were asked to sketch in the legs of a bumblebee at the correct position on the body—a problem that was solved incorrectly by 97.6% of students. Only 2.4% of participants (*N* = 812) sketched three legs from the thorax. The majority of students (68.6%) knew that bumblebees have three pairs of legs; however, they did not know that all six legs derive from the thorax. A total of 28.8% of students sketched in more or fewer than three legs. Typical examples of sketches are shown in Figure 1. 

#### 3.1.3. Ecology and Agricultural Importance

Participants (*N* = 866) correctly answered 54.95% (*SD* = 25.31%) of questions in the category ‘ecology and agricultural importance’. The majority (79.9%) of students (*N* = 860) knew that bumblebees live in a close relationship with plants, similar to honeybees (E2, Table 4). In contrast, 53.8% of students (*N* = 856) did not know whether the pollination of crops by bumblebees can lead to yield increases (E1, Table 4). Less than half (40.3%) knew that pollination by bumblebees can increase crop yield. Furthermore, well over one-quarter (33.5%) of students (*N* = 849) did not know whether some bumblebee species in Germany are threatened with extinction. In contrast, 61.4% of participants knew that some bumblebee species are threatened with extinction.

#### 3.1.4. Colony Life

Overall, students (*N* = 867) correctly answered 26.67% (*SD* = 17.87%) of questions in the category ‘colony life’. 61.9% of students (*N* = 855) did not know that a bumblebee colony consists mainly of females (C1, Table 4). The correct answer was given by 15.7% of participants and the wrong answer by 22.5%. In contrast, 64.7% of students (*N* = 861) knew that members of a bumblebee colony undertake different tasks (C2, Table 4); 30.3% of students did not know whether this is true and 5% of students answered the question incorrectly.

#### 3.1.5. Species Identification

**Figure 2:**
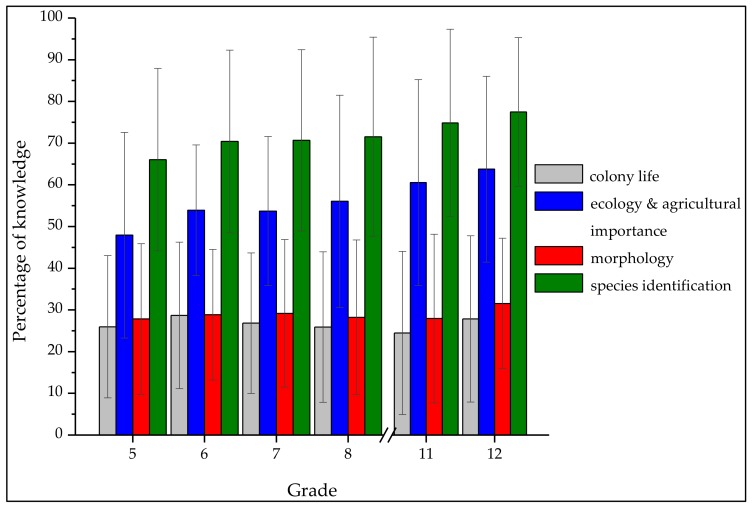
Mean (*SD* = error bars) of knowledge about bumblebees in the four categories (grey = colony life; blue = ecology and agricultural importance; red = morphology; green = species identification) of knowledge in different grades.

In the category ‘species identification’, students had to decide whether a bumblebee was shown in the color picture provided. Overall, students answered this category best (Figure 2): 71.25% (*SD* = 22.01%) of questions in the category species identification were answered correctly by students (*N* = 867). Regarding item P1, 90.1% of participants (*N* = 857) identified the buff-tailed bumblebee *Bombus terrestris* as a bumblebee in the picture (Table 4) and 92.8% of students (*N* = 817) knew that the depicted wasp *Vespula germanica* was not a bumblebee. Nevertheless, students had difficulty in distinguishing between bumblebees and another wild bee (E3, Table 4). Thus, 71.2% of students (*N* = 848) thought that the hornfaced bee *Osmia cornuta* is a bumblebee. Only 12.1% of participants knew that *Osmia cornuta* is not a bumblebee. 

Figure 2 shows the percentage of correct answers per category for different grade levels. Since the four categories were assigned a different number of questions, the correct answers are given as percentages.

#### 3.1.6. Differences and Correlations of Knowledge in Different Grades

We found the following significant differences in knowledge between grade levels: knowledge in the category ‘ecology & agricultural importance’ differed significantly between students of grade five (*Mdn* = 42.86%) and students of grade eight (*Mdn* = 57.14%, *U* = 8973.5, *z* = −3.2, *p* = 0.001, *r* = 0.18), eleven (*Mdn* = 71.43%, *U* = 7,017, *z* = −4.22, *p* < 0.001, *r* = 0.25) and twelve (*Mdn* = 71.43%, *U* = 3704, *z* = 4.49, *p* < 0.001 *r* = 0.29). 

Students of grade five (*Mdn* = 66.67%) also recognised significantly fewer bumblebees in pictures than students of grade eleven (*Mdn* = 83.33%, *U* = 7107.5, *z* = −4.18, *p* < 0.001, *r* = 0.25) and twelve (*Mdn* = 83.33%, *U* = 3971.5, *z* = −3.92, *p* < 0.001, *r* = 0.25). The detected effects within the categories ‘ecology and agricultural importance’ and ‘species identification’ were small [63]. The greatest difference in the category ‘ecology and agricultural importance’ was detected between grade five and grade twelve, with a mean of 1.1 more correctly answered questions out of seven. The greatest difference in the category ‘species identification’ was detected between 5th grade students and 12th grade students and amounted to a mean of 1.4 more correctly answered questions out of twelve. There were no significant differences between the grades in the categories ‘colony life’ and ‘morphology’. 

Considering all the correctly answered questions of knowledge, students of grade five (*Mdn* = 42.86%) knew significantly less than students of grade eleven (*Mdn* = 51.43%, *U* = 7486, *z* = −3.49, *p* < 0.001, *r* = 0.21) and twelve (*Mdn* = 54.29%, *U* = 3545.5, *z* = −4.77, *p* < 0.001, *r* = 0.31). The effects of differences were small to medium [63]. On average, a student of grade twelve could answer 2.9 questions more (out of 35) than a 5th grade student. This indicates a very small learning progress between 5th grade students and students of German senior grades (grades eleven and twelve).

The grade of the students was significantly related to knowledge in the category ‘ecology & agricultural importance’ (*r* = 0.19, *p* < 0.001) and to the knowledge in the category ‘species identification’ (*r* = 0.18, *p* < 0.001). The detected correlations had small effects [63].

### 3.2. Attitude

Attitude was measured by a five-tier Likert-scale (Table 2). A higher numeric value (min = 1; max = 5) correlates with a more positive attitude towards bumblebees. The students’ attitude (*N* = 866) was slightly positive, with a mean score of 3.65 (*SD* = 0.80) (see Table 5). The attitude was significantly correlated with the grade (*r* = −0.21, *p* < 0.001), i.e., the higher the grade, the more negative was the attitude to bumblebees of the participant.

We found significant differences between grades considering the attitude in total. The attitude of grade five (*Mdn* = 4.00) differed significantly to that of grade eight (*Mdn* = 3.50, *U* = 7868, *z* = −4.61, *p* < 0.001, *r* = 0.26) and eleven (*Mdn* = 3.41, *U* = 6138, *z* = −5.53, *p* < 0.001, *r* = 0.33). Students from grade six (*Mdn* = 3.82) had a significantly more positive attitude than those from grade eleven (*Mdn* = 3.41, *U* = 7354.5, *z* = −3.24, *p* = 0.001, *r* = 0.19). A significant difference in attitude was also observed from grade seven (*Mdn* = 3.82) to grade eleven (*Mdn* = 3.41, *U* = 6787.5, *z* = −3.75, *p* < 0.001, *r* = 0.23).

#### 3.2.1. Differences and Correlations in Attitude between Different Grades

**Figure 3:**
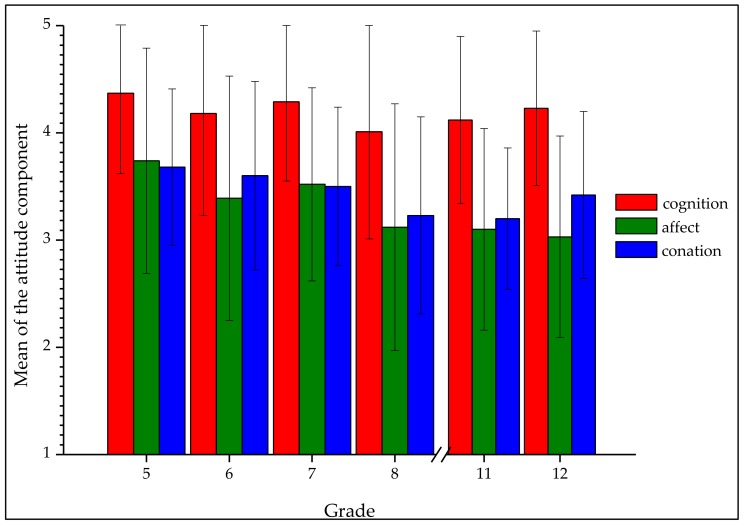
Mean (*SD* = error bars) of the subscales of the attitude (red = cognition; green = affect; blue = conation) towards bumblebees in different grades.

We found significant differences concerning the components of the attitude and grade. The mean attitude components for each respective grade is shown in Figure 3.

#### 3.2.2. Cognition

Regarding the cognitive component of the attitude, students (*N* = 865) were on average positive-thinking (*M* = 4.21; *SD* = 0.83) (Figure 3). We found a significant negative correlation between grade and the cognitive component (*r* = −0.12, *p* = 0.001). Grade five (*Mdn* = 4.6) differed significantly from grade eight (*Mdn* = 4.2, *U* = 9092, *z* = −3.04, *p* < 0.002, *r* = 0.17) and eleven (*Mdn* = 4.4, *U* = 7684.5, *z* = −3.33, *p* < 0.001, *r* = 0.20). The cognition component had the highest mean and lowest standard deviation of the three components of attitude (Figure 3). Both detected differences had small effect sizes [63]. The results indicate that the cognitive rating for bumblebees by students is very positive. On average, students rated the cognitive component of the attitude only 0.79 lower than the maximum.

#### 3.2.3. Affect

Considering the affective component of the attitude (*N* = 866), the mean value was *M* = 3.37 (*SD* = 1.06). Younger students had a more positive attitude than older ones. Affect was significantly negatively correlated with the grade (*r* = −0.23; *p* < 0.001). The affective component of the attitude differed significantly between students of grade five (*Mdn* = 4.0) and grade six (*Mdn* = 3.4, *U* = 11,988.5, *z* = −2.88, *p* = 0.004, *r* = 0.16), eight (*Mdn* = 3.2, *U* = 7732.5, *z* = −4.79, *p* < 0.000, *r* = 0.27), eleven (*Mdn* = 3.0, *U* = 6196.5, *z* = −5.45, *p* < 0.001, *r* = 0.32), and twelve (*Mdn* = 3.2, *U* = 3423, *z* = −5.02, *p* < 0.001, *r* = 0.32). A significant difference was also observed in the affective component of the attitude between students of grade seven (*Mdn* = 3.6) and those of grade eleven (*Mdn* = 3.0, *U* = 6735.5, *z* = −3.84, *p* < 0.001, *r* = 0.23) and twelve (*Mdn* = 3.2, *U* = 3728, *z* = −3.73, *p* < 0.001, *r* = 0.25). The affective component of attitude towards bumblebees had the lowest mean and highest standard derivation of all three components of attitude. The detected effect sizes were small to medium [63]. Overall, students demonstrated a moderately positive perception of the affective component of attitude (Figure 3).

#### 3.2.4. Conation

The mean value for the category conation of attitude was *M* = 3.46 (*SD* = 0.99). Conation was significantly negativly correlated with the grade (*r* = −0.16; *p* < 0.001). Grade five (*Mdn* = 3.83) differed significantly from grade eight (*Mdn* = 3.29, *U* = 8532.5, *z* = −3.74, *p* < 0.001, *r* = 0.21) and eleven (*Mdn* = 3.29, *U* = 6990, *z* = −4.30, *p* < 0.001, *r* = 0.25). Also in the category conation, grade six (*Mdn* = 3.71) differed significantly to grade eight (*Mdn* = 3.29, *U* = 8524, *z* = −3.18, *p* = 0.001, *r* = 0.18) and grade eleven (*Mdn* = 3.29, *U* = 7043.5, *z* = −3.71, *p* < 0.001, *r* = 0.22). The mean of the behavioural component of attitude was similar to the affective component in different grades. All detected effect sizes of differences were small. Overall, students had a moderately positive perception of attitude’s conation (Figure 3).

There were no significant differences between the other grades. 

## 4. Discussion

### 4.1. Knowledge about Bumblebees

Our results support the findings of other authors that students only know few facts about insects [34,35,36,37,38,40,41,42,43]. Overall, students in grades five to twelve knew very little about bumblebees. Moreover, the mean knowledge values differed only minimally between the grade levels (Table 4). Relating to our first research question, we can conclude that students are not familiar with the biology of bumblebees. The high standard deviation of the mean of total knowledge and especially of different categories of knowledge, indicate major differences in the level of knowledge within a single grade level. This suggests that students possibly acquire knowledge in their leisure time independent of school education [51]. The amount of leisure study might differ considerably between students in the same grade and might explain the large differences observed within the grades.

Another potential explanation for the large observed differences in knowledge between students within the same grade level might relate to existing knowledge of other biological facts. For example, students might transfer knowledge about other social insects to bumblebees.

#### 4.1.1. Morphology

In total, the category ‘morphology’ was answered the second worst of all four knowledge categories. This might be because most of the questions can only be answered following very careful observation of bumblebees. For example, most students could not draw the legs on the correct body part of a bumblebee (M2, Table 2). The authors in [34] also assessed whether students can sketch legs on ant bodies in the appropriate position. They determined that most students were familiar with the number of legs, but could not draw them at the correct position, a finding supported by our data here. Nevertheless, 19% could draw the legs on the correct position of an ant [34], but only 2.4% were able to draw the legs on a bumblebee correctly. An explanation for this difference might be due to the three-dimensional locomotion of bumblebees. Worker ants do not have wings and therefore, can only move in two-dimensional space. Thus, ants can be observed without them fearing being approached. Therefore, students might observe ants in more detail than bumblebees in nature. A further reason for the lack of knowledge concerning insect leg positioning might be that the origin of insect legs can best be observed if the insect is observed from the side or from below. This is generally not the case in the natural environment, in which insects are normally observed from above. For bumblebees, wings and hair obscure the view of the origin of their legs. However, because the number of legs can be accurately and easily detected by the observation of living insects, it is not surprising that this is known by students. Another possible explanation might be that insect illustrations in German schoolbooks often focus on their internal structure, such as the tracheal or nervous systems and their external morphology is neglected [24,25]. Additionally, many students may be familiar with insects and honeybees, in particular, from cartoon books and animated television series such as ‘Die Biene Maja’ (engl. ‘Maya the Bee’) which is a based upon a children’s book from the 1920s by Waldemar Bonsels. Here, insect anatomy follows a general principle with two of six legs located at the abdomen [32]. Therefore, a reason for insufficient morphological knowledge might be that students focus on other aspects of insects in biology lessons. For example, honeybees are a common topic in German biology education, but mostly in the context of pollination [18,19,23,25,26,64,65,66]. Limited knowledge of insect morphology by students is also reported by Barrow [39].

#### 4.1.2. Ecology and Agricultural Importance

Overall, students could answer slightly more than half of the questions in the category ‘ecology and agricultural importance’ correctly. This might be because most questions in this category focused on pollinators in general and did not require much specialist knowledge, coupled with the fact that pollination is a perennial part of German biological education. Nevertheless, the responses within the category ‘ecology and agricultural importance’ had the highest standard deviation of all categories of knowledge. This category represents over one-quarter of all questions, suggesting that large differences in knowledge exist in this field. Regarding item E2 (Table 2), most students knew that bumblebees live in a close relationship with plants. This knowledge is very general but is important to reach conclusions and offer ideas concerning connections within ecosystems. The reason for the high percentage of correct answers to this question might be that this fact can be observed in nature very easily; therefore, students could rely on their direct experiences in nature to provide the correct answer. The results of item E1 (Table 2) indicate that many students are aware that bees are threatened. This might be due to the great media presence of bee mortality. However, well over a quarter of students did not know that some native bumblebee species are threatened with extinction in Germany. Without knowledge that animal species are under threat of extinction, the impetus is lacking to rethink lifestyle changes and their associated effects on the respective organism. Therefore, we must improve student knowledge about specific insect species and their endangerment. Item E3 (Table 2) deals with the agricultural importance of pollination by bumblebees. Because pollination results in an increase in the agricultural output of some crops, it is economically important and knowledge about it clearly illustrates our human dependence on pollinators. Therefore, it is necessary to raise the students’ awareness of it. To answer this question correctly, students might have transferred their knowledge about pollination from honeybees to bumblebees. Pollination by honeybees is a common example in German schoolbooks [23,25]. 

#### 4.1.3. Colony Life

The item with the highest percentage of correct answers out of all twelve items within the category ‘colony life’ was item C2 (Table 2). Most students knew that members of a bumblebee colony undertake different tasks. On the one hand, it is encouraging that slightly less than two-thirds of students knew the correct answer; however, if we consider that division of labor is a fundamental basis of all social insect colonies, then almost one-third of students did not know this piece of general knowledge about colony life. Knowledge about the division of labor might be transferred to bumblebees from other social insects, such as honeybees. In total, students knew least about the category ‘colony life’, indicating that this topic must become a greater focus in biological education in Germany in the future.

#### 4.1.4. Species Identification

Altogether, the category ‘species identification’ was answered on average the best out of the four categories of knowledge. On average 71.25% of questions were answered correctly. As to our second research question, our data show that many students are capable of differentiating bumblebees from other insects depicted in photographs. However, the responses also showed a high standard deviation (Figure 2). Items P1 and P2 were answered correctly by most students. The percentage of correct answers was very high (Table 2). The buff-tailed bumblebee, *Bombus terrestris*, and the wasp *Vespula germanica*, are common in Germany and students have probably encountered both insects in their daily lives. Our results are in line with Wilson, Forister and Carril [44]. Nevertheless, item P3 led to the finding that most students confused the hornfaced bee *Osmia cornuta* with a bumblebee. *Osmia cornuta* is also common in Germany. *Osmia cornuta* resembles a bumblebee: its hairy coat gives it a sturdy appearance and perhaps reminds students of a bumblebee. Students might therefore connect bumblebees with a hairy appearance. To improve the ability to distinguish bumblebees and other wild bees and to strengthen students’ appreciation of the diversity of nature, the diversity of insect species should be a stronger focus in school in the future.

#### 4.1.5. Knowledge in Different Grades

We did not detect any significant differences in knowledge in the categories colony life and morphology. Prokop, Prokop and Tunnicliffe [36] also found no evidence that older children had better knowledge concerning the invertebrate respiratory system than younger children. Barrow [39] instead found a broader understanding of insect characteristics in older children. We could not confirm any correlations or differences between grade levels in the category ‘morphology’. This difference between our study and other studies might be because they evaluated younger children’s knowledge [36,39]. The absence of significant differences in the categories ‘morphology’ and ‘colony life’, together with the mean knowledge scores in these categories (Figure 2), indicate that students do not learn about these in secondary school. Therefore, more information should be provided in the future.

We detected differences in the categories ‘ecology and agricultural importance’, ‘species identification’, and ‘total knowledge’. All detected correlations and differences in these three categories between grade levels had small-to-medium effect sizes. Nevertheless, the observed differences between grade levels indicate that the lack of knowledge is greater in more senior grades; however, a significant positive correlation between knowledge and grade was observed and thus, our results agree with those of other researchers. The authors in [36] also analyzed the age and knowledge of participants and found that higher grades had a greater degree of knowledge. 

The effect sizes detected here indicate a very small increase in bumblebee knowledge in German secondary schools. One reason for this might be that biology teachers do not address the topic in class. If teachers were to introduce the topic of bumblebees into school teaching, knowledge would subsequently increase after the grade where the topic was introduced, resulting in differences between grades. Therefore, if bumblebees are not dealt with in biology lessons, students have to assimilate knowledge about bumblebees during their leisure time or via the transfer of knowledge from other insects and pollinators to bumblebees. However, transferred knowledge is unsatisfactory to answer specific questions, for example, whether a bumblebee can fend off an enemy by stinging (see M1; Table 2). One reason why bumblebees are not included in school curricula might be that the biology curricula in the German states of Rhineland-Palatinate and Hesse are worded in an open way. For example, curricula are forced to address the topic ‘interactions in an ecosystem’. However, the type of ecosystem, i.e., whether plant or animal, is not prescribed [31,67]. Therefore, biology teachers themselves can decide which animals and plants they exemplify and they might use examples from schoolbooks and existing teaching material. The fact that schoolbooks promote similar flagship species, might lead to an underappreciation of biodiversity. The honeybee is usually used as a flagship species to illustrate pollination and the life of a social insect [18,19,23,25,26,64,65,66], but teaching material about wild bees is highly underrepresented. One opportunity to change the existing situation might be to provide innovative educational material about less commonly known genera, such as bumblebees, to biology teachers, thereby presenting students with a greater variety and range of nature.

Students possess little knowledge about bumblebees. This situation should put us on alert, because a willingness to behave in an ecofriendly way is influenced by knowledge [14,15,32]. If students are not aware that some native bumblebees are threatened with extinction [11], they might not recognize the need to protect them. Without knowledge for example, about the food sources and habitat of bumblebees, students cannot imagine conservation opportunities. If we consider the loss of bees and insects, we recognize the absolute importance of increasing knowledge transfer about insects in German biology education. To close the demonstrated gap of knowledge concerning bumblebees, German biology education should focus on this topic in the future.

### 4.2. Attitude towards Bumblebees

Attitude towards bumblebees of all grade levels was in the positive range (Table 5). One reason for this might be a positive social perception of bumblebees. This is well-illustrated by a German proverb, in which bumblebees are depicted as an active, enterprising, and alert organism [68] (p. 69).

We found significant differences between grade levels in all components of attitude, similar to those for the attitude in total. The attitude in total differed significantly between grades five and eight, five and eleven, six and eleven, and seven and eleven. All effect sizes were small to medium. Furthermore, the correlation between grade level and attitude had a small effect. Strikingly, positivity of attitude was correlated with student grade, with younger students having a more positive attitude, which became more negative with increasing grade. This is in line with other studies about attitude towards honeybees [48]. A reason for this might be that younger students have a more positive attitude towards science in general [69]. Moreover, studies have shown that younger students are more interested in animals and plants while older students are interested in the human biology [70,71]. Again, the attitudes towards the study of biology are closely related to interest [72].

Large standard deviations within the attitude of one grade level probably partly result from the construct of the attitude itself (Table 5 and Figure 3). The attitude is an individual characteristic that does not have to be the same within an age group; nevertheless, the mean and the standard deviation are instructive in comparisons between grade levels.

The three components of attitude also differed significantly between grades, but all the observed differences were small to medium. The complete attitude towards bumblebees is also reflected in all three sub-components: students of lower grades had a more positive attitude than those of higher grades. Notably, for the components in general, the mean of cognition was more positive than those the other two components, affect and conation, which were only moderately positive (Figure 3). Therefore, it appears that students have an essentially positive attitude, but affect and conation could be improved.

Our results of student attitude towards bumblebees do not correspond with findings of others on attitude towards other insects. In previous studies, insects have been associated with fear and dislike [33,37,47,48] and bees, especially, have been associated with fear [27,28]. However, bumblebees might be an exception, which makes them optimal flagship species for environmental education and conservation [49,50]. The positive attitude of students represents a prerequisite for environmentally friendly behavior [14,15,32], although affect and conation could be enhanced, indicating that students’ feelings, as well as their behavioral inclinations and intentions towards bumblebees could be improved. Conation in particular, should be more of a focus if students are to make a greater contribution to environmental protection. One great opportunity to influence students’ attitude might be the accumulation of direct experiences for example by observing bumblebees in biology lesson [53,73,74,75].

We have to take into account that the attitude could worsen if students know more about bumblebees, e.g., that they can sting. Further research should draw attention to it.

In terms of methodical aspects of attitude, this study presents a reliable and valid instrument with which to measure students’ attitude towards bumblebees and the internal consistency was good (α = 0.90). This was the first time that we tested this instrument. The dimensional structure was verified by a principal component analysis. We detected high loadings on two components at items V10, V15, V17 (Table 2), but because of logical explanations for these cross-loadings, we decided to retain these items. For example, item V17 (‘If I see a bumblebee in my room, I would kill it.’) was highly loaded by cognition and conation. However, it is logical that you perform the operational intent because of your beliefs and thoughts that bumblebees are useful organisms and that killing them is the wrong way to remove them from a room. Therefore, we placed the item into the category conation. In the future, we should retain the contextual topics of the relevant items with high cross-loadings, but attempt to formulate them more specifically.

### 4.3. Limitations of the Study

Firstly, we did not select the participants randomly; they were students of classes of cooperating teachers. This resulted in no 9th-grade students, which meant that this grade could not be compared with other grades. Secondly, the knowledge questionnaire was constructed to include simple and difficult questions within each category. Even though the perceived difficulty level varied, we could not interrogate every aspect of knowledge. For example, no question was related to the internal organs or the respiration of bumblebees. Nevertheless, the questionnaire was tested in a pilot study prior to this study and we applied the construct of face validity. Thirdly, the questionnaire of attitude was applied for the first time. As depicted before, it should retain the contextual topics, but highly cross loaded items should be formulated more specifically in the future.

## 5. Conclusions and Further Research

The decline of insects and loss of biodiversity is currently a big problem [1,2,6,9,10]. Biological education could play a role in influencing thinking and behavior [17]. Therefore, students need to know much about insects and to have a positive attitude towards them [14,15,32]. We studied the knowledge and attitudes of students to bumblebees because we imagined bumblebees to meet these requirements.

Our results reveal gaps of knowledge, but also a positive attitude of students towards bumblebees. To close these gaps of knowledge, German biology education should focus on bumblebees in the future. To aid biology teachers to focus on insects such as bumblebees, innovative educational material should be developed and distributed to them. Based on this, the willingness to enact environmentally friendly and the willingness to conserve bees and other insects will be increased. One innovative way to do this might be to teach concepts that enable the accumulation of direct experiences [38,73,75,76].

The attitude of students towards bumblebees was positive, which makes them optimal flagship species for environmental education and conservation; nevertheless, affect and conation could be improved. Conation in particular, should become a greater focus if we want to encourage students to positively influence environmental protection.

Our results should be used to develop teaching materials adapted to knowledge and attitudes in the future. This study provides a solid basis for developing innovative materials and methods that neither overwhelm nor overburden students. Therefore, it is feasible to fill in knowledge gaps. This could be an approach to increase sensitivity to the decline of insects in biology lessons and to inspire students to become conservationists.

## Figures and Tables

**Table 1:** Descriptive statistics (discrepancies exist between the total number and the sum of males and females because not all participants stated their gender). Grade ten and 13 were excluded of grade comparison because of the small sample sizes.

	5th Grade	6th Grade	7th Grade	8th Grade	10th Grade	11th Grade	12th Grade	13th Grade
**Age in years**	9–12	10–12	11–13	12–15	14–16	15–17	16–19	17–20
**Male**	96	85	83	61	8	47	28	25
**Female**	75	77	73	68	6	64	38	12
**Total**	175	168	162	130	14	114	67	37

**Table 2:** Measurement and exploratory factor analysis of the attitude towards bumblebees. The questionnaire was originally in German. All items have been translated into English for this article. For the analysis, the statements marked with a (−) have already been inverted. Factor loadings below 0.40 are not included.

Variable		Rotated Factor Loadings		
		Cognition	Affect	Conation
V01	I think bumblebees are unnecessary. (−)	0.75	-	-
V02	Bumblebees are ‘vermin’ and I could dispense with them. (−)	0.74	-	-
V03	Bumblebees are useful animals. (+)	0.69	-	-
V04	Bumblebees are an important part of our environment. (+)	0.69	-	-
V05	Bumblebees are lazy. (−)	0.58	-	-
V06	I think bumblebees are exciting. (+)	-	0.82	-
V07	I think bumblebees are appealing/interesting. (+)	-	0.80	-
V08	I think bumblebees are fascinating. (+)	-	0.80	-
V09	I would like to observe living bumblebees at school. (+)	-	0.66	-
V10	If I think of bumblebees, I connect them with good things. (+)	0.46	0.51	-
V11	It would bother me very much if a bumblebee were near me or were even to land on me. (−)	-	-	0.74
V12	If a bumblebee lands on me, I would stay calm. (+)	-	-	0.73
V13	I would observe bumblebees only if they sit in a box and cannot fly to me. (−)	-	-	0.71
V14	If I would discover a bumblebee in my room, I would look at it more closely. (+)	-	-	0.64
V15	If a bumblebee lands on me, I would watch it carefully. (+)	-	0.44	0.63
V16	If I discover a bumblebee in my room, I would put it outside. (+)	-	-	0.44
V17	If I see a bumblebee in my room, I would kill it. (−)	0.46	-	0.41
Eigenvalues		6.24	1.90	1.35
% of variance		36.71	11.16	7.96
Cronbach’s α		0.79	0.85	0.82

**Table 3:** Mean scores of total knowledge as a percentage. Increasingly higher values are correlated with an increase in knowledge by students about bumblebees.

Grade		5	6	7	8	11	12
**Total Knowledge in %**	***M***	44.47	47.98	47.71	48.04	49.58	52.79
	***SD***	13.26	13.07	12.59	14.41	14.58	12.88

**Table 4:** Test on knowledge about bumblebees. The questionnaire was originally in German; items have been translated into English for this article. The percentage of the answers represents the mean for all participants. Items M1, E1, E2, E3, C1, C2, P1, P2 and P3 are closed questions where participants had to tick one answer out of the presented answers.

Category	Item			Possible Answers	Right Answer (%)	Wrong Answer (%)	Do Not Know (%)
**Morphology**	M1	To fend off enemies, bumblebees…		bite stingbite and sting neither bite or sting do not know	21.8	66.1	12.1
	M2	Sketch in all the legs of the bumblebee in the picture, please.		sketch in 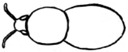	2.4	90.9	6.7
**Ecology and agricultural importance**	E1	Some native bumblebee species are threatened with extinction in Germany.		yes no do not know	61.4	5.2	33.5
	E2	Like honey bees, bumblebees live in a close relationship with plants.		yes no do not know	79.9	2.9	17.2
	E3	The pollination of crops by bumblebees can lead to yield increases.		yes no do not know	41.0	4.3	54.7
**Colony life**	C1	There are mainly male insects in a bumblebee colony.		yes no do not know	22.5	15.7	61.9
	C2	Members of a bumblebee colony undertake different tasks.		yes no do not know	64.7	5.0	30.3
**Species identification**	P1	Please tick, if there is a bumblebee in the picture.	*Bombus terrestris* *	yes no do not know	90.1	4.8	5.1
	P2		*Vespula germanica* *		92.8	2.4	4.8
	P3		*Osmia cornuta* *		12.1	71.2	16.6

* The questionnaire comprised 12 color photographs. Participants had to tick *yes* (= it is a bumblebee), *no* (= it is not a bumblebee) or *do not know* (= I am not sure whether it is a bumblebee or not).

**Table 5:** Mean of the attitude towards bumblebees in the different class levels. The numeric value of the attitude scale ranges from one to five. A higher value represents a more positive attitude.

Class Level		5	6	7	8	11	12
**Attitude in total**	***M***	3.90	3.71	3.74	3.42	3.44	3.54
	***SD***	0.73	0.88	0.74	0.92	0.66	0.78
